# Bortezomib Reduces the Tumorigenicity of Multiple Myeloma via Downregulation of Upregulated Targets in Clonogenic Side Population Cells

**DOI:** 10.1371/journal.pone.0056954

**Published:** 2013-03-04

**Authors:** Miho Nara, Kazuaki Teshima, Atsushi Watanabe, Mitsugu Ito, Keiko Iwamoto, Atsushi Kitabayashi, Masaaki Kume, Yoshiaki Hatano, Naoto Takahashi, Shinsuke Iida, Kenichi Sawada, Hiroyuki Tagawa

**Affiliations:** 1 Department of Hematology, Nephrology, and Rheumatology, Akita University Graduate School of Medicine, Akita, Japan; 2 Department of Internal Medicine, Akita Kumiai General Hospital, Akita, Japan; 3 Department of Internal Medicine, Hiraka General Hospital, Yokote, Japan; 4 Department of Internal Medicine, Yamamoto Kumiai General Hospital, Noshiro, Japan; 5 Department of Medical Oncology and Immunology, Nagoya City University School of Medical Science, Nagoya, Japan; Virginia Commonwealth University, United States of America

## Abstract

Side population (SP) cells in cancers, including multiple myeloma, exhibit tumor-initiating characteristics. In the present study, we isolated SP cells from human myeloma cell lines and primary tumors to detect potential therapeutic targets specifically expressed in SP cells. We found that SP cells from myeloma cell lines (RPMI 8226, AMO1, KMS-12-BM, KMS-11) express CD138 and that non-SP cells include a CD138-negative population. Serial transplantation of SP and non-SP cells into NOD/Shi-scid IL-2γnul mice revealed that clonogenic myeloma SP cells are highly tumorigenic and possess a capacity for self-renewal. Gene expression analysis showed that SP cells from five MM cell lines (RPMI 8226, AMO1, KMS-12-BM, KMS-11, JJN3) express genes involved in the cell cycle and mitosis (e.g., *CCNB1, CDC25C, CDC2*, *BIRC5, CENPE, SKA1*, *AURKB, KIFs*, *TOP2A, ASPM*), polycomb (e.g., *EZH2, EPC1*) and ubiquitin-proteasome (e.g., *UBE2D3, UBE3C, PSMA5*) more strongly than do non-SP cells. Moreover, *CCNB1, AURKB*, *EZH2* and *PSMA5* were also upregulated in the SPs from eight primary myeloma samples. On that basis, we used an aurora kinase inhibitor (VX-680) and a proteasome inhibitor (bortezomib) with RPMI 8226 and AMO1 cells to determine whether these agents could be used to selectively target the myeloma SP. We found that both these drugs reduced the SP fraction, though bortezomib did so more effectively than VX-680 due to its ability to reduce levels of both phospho-histone H3 (p-hist. H3) and EZH2; VX-680 reduced only p-hist. H3. This is the first report to show that certain oncogenes are specifically expressed in the myeloma SP, and that bortezomib effectively downregulates expression of their products. Our approach may be useful for screening new agents with which to target a cell population possessing strong tumor initiating potential in multiple myeloma.

## Introduction

Multiple myeloma (MM) is characterized by the accumulation of a population of malignant plasma cells (10% and the more) within the bone marrow [1], [2]. It is the second most frequently occurring hematological disease, affecting mainly elderly individuals [2], and is diagnosed through blood tests (serum protein electrophoresis, serum free kappa/lambda light chain assay), bone marrow examination, urine protein electrophoresis, and X-ray of commonly involved bones. MM is generally responsive to conventional chemotherapy followed by myeloablative doses of alkylating agents and autologous stem cell transplantation [2], [3]. However, cytotoxic chemotherapy-based treatment is not curative, and the disease eventually recurs [2], [4]–[6]. This is in part because although currently available anti-MM strategies effectively target the bulk of tumor cells, they do not target the tumor-initiating subpopulation (i.e., cancer stem cells). The obvious need for new approaches to the treatment of MM has provided an incentive for the rapid bench-to-bedside translation of new drug treatments, including the proteasome inhibitor bortezomib, aurora kinase inhibitors and immunomodulatory drugs (IMiDs) such as thalidomide and lenalidomide, as well as novel therapies such as stem cell transplantation [2], [4]–[7].

Side population (SP) cells are identified based on their ability to export Hoechst 33342 dye via an ATP-binding cassette (ABC) membrane transporter, which gives these cells a distinct low-staining pattern with Hoechst 33342 [8]–[14]. SP cells exhibit key characteristics of cancer initiating cells, including capacities for differentiation, repopulation, clonogenicity and self-renewal [8]–[15]. They also express high levels of various members of the ABC transporter family, including ABCB1 (MDR1/P-glycoprotein) and ABCG2 (MXR/BCRP), which in addition to inhibiting Hoechst staining, is responsible for the cells’ drug resistance [8], [17]. Given that many different types of cancer cells overexpress both of these ABC transporters, it seems reasonable to screen for stem-like fractions among cancer cells based on this characteristic. The SPs from various MM cell lines were recently investigated by Jakubikova and coworkers [18]. They found that MM SP cells exhibit cancer stem cell-like characteristics and that they have greater tumorigenic potential than non-SP (main population; MP) cells. This suggests there is a difference in the gene expression profiles of SP and MP cells. However, there have as yet been no reports of genes specifically expressed in the myeloma SP. In the present study, therefore, we investigated the genes and gene products that were up- or downregulated in myeloma SP cells. Our aim was to identify candidate therapeutic targets expressed within the myeloma SP. For this purpose, we initially endeavored to determine whether myeloma SP cells exhibit cancer-initiating characteristics.

## Materials and Methods

### Primary MM Samples

SP and MP cells from eight cases of plasma cell myeloma and one case of plasma cell leukemia were collected from Akita University Hospital, Yamamoto Kumiai General Hospital, Akita Kumiai General Hospital and Hiraka General Hospital. This study was conducted with written informed consent of the study participants and the approval of these Institutional Review Boards, according to the Declaration of Helsinki prior to collection of the specimens. Six samples of primary tumor cells were obtained from freshly isolated bone marrow at the time of diagnosis; two other samples (M4 and M8) were obtained after the first melphalan-prednisolone treatment showed no chemotherapeutic response. Primary tumors cells were purified from freshly isolated bone marrow of MM patients by Ficoll-Hypaque density sedimentation. Aspirated myeloma cell from bone marrow, and mononuclear cells were harvested in RPMI 1640 (+20%FBS) with 15% DMSO and stocked in Liquid nitrogen tank for cell preservation. For SP analysis, cells were cultured in RPMI-1640 containing 10% heat-inactivated FBS, 100 U/mL penicillin, 100 µg/mL streptomycin and 2 mM L-glutamine, and were maintained at 37°C in 5% CO_2_. Information about the patient samples is summarized in Table 1.

**Table 1:** Information of primary MM samples.

MM no.	age	sex	tumortype	SPanalysis	Serum Mprotein	FISH#4	p53 FISH	Therapybefore R#3	MMno.	Karyotype (%)
M1	62	F	PCM#1	at ID#2	IgG κ	4p16(FGFR) gain#5	Del#7 17p13.1(38%)	–	M1	#9
M2	82	M	PCM	at ID	IgG λ	14q32 gain	–	–	M2	#10
M3	54	M	PCM	at ID	IgA λ	14q32 gain	–	–	M3	#11
M4	48	F	PCM	at R#3	IgG λ	ND#6	–	MP#8	M4	46,XX
M5	59	M	PCM	at ID	IgG λ	–	–	–	M5	46,XY
M6	65	F	PCM	at ID	IgG κ	ND	–	–	M6	46,XX
M7	82	F	PCM	at ID	IgG λ	–	–	–	M7	46,XX
M8	86	F	PCM	at R	IgG λ	ND	–	MP#8	M8	#12

#1 PCM: plasma cell myeloma.

#2 ID: Initial Diagnosis.

#3 R: Recurrence.

#4 FISH: fluorescence in situ hybridization (100 count).

#5 gain: DNA copy number gain.

#6 ND: Not Done.

#7 del (deletion): DNA copy number loss.

#8 MP: melphalan+prednisolone.

#9 61,X,-X,-X,t(1;18)(p13;q21),+5,−8, −10, −12, −13, −14, +15, −16, add(17)(p11.2), −18,+19, −20, −22[5%].

#10 45,X,der(Y)t(Y;1)(q11.2;q12),add(1)(p13),add(5)(p15), −13, −14,+der(?)t(?;7)(?;q11.2);2/20; 46,XY [85%]. ^#11^58<2n>,XY,+3,+5,+del(5)(q?),+6,+7,+8,+9,+10,+18,+19,+mar1[10%]/46,XY[90%]%].

#12 43,XX,t(6;14)(p21;q32), −13,?t(14;16)(q32;q23), −16, −22 [4]; 46,XX[80%].

### MM Cell Lines

We used 14 MM cell lines [18]–[21], including RPMI 8226, KMS-11, JJN3, NCI-H929, U266, KMS-12-BM [20], KMS-5 [20], AMO1 [21], XG7 [22], FR4 [23], PCM6 [24], KMS-20 [25], KMS-26 [25] and KMS-27 [26] cells. Among them, RPMI8226, NCI-H929, U266, KMS-11 and JJN3 cell lines were obtained from American Type Culture Collection (ATCC).

MM cell lines of KMS-5, KMS-11, KMS-12-BM, KMS-20, KMS-27 were obtained from Dr. Ohtsuki, Kawasaki Medical University, Japan), RPMI 8226 NCI-H929, U266 and JJN3,were from American Type Tissue Collection. PCM6 was obtained from Riken BioResource Center (Japan). FR4 was kindly provided from Dr. Tagawa, Osaka City University Medical School; AMO1 from Dr Shimizu, Shimane Prefectural Central Hospital, and XG7 from Dr Klein, Institute for Molecular Genetics, Montpellier, France. MM cell lines were cultured in RPMI 1640 (Cellgro, Mediatech, VA) supplemented with 10% heat-inactivated fetal bovine serum (FBS; Harlan, Indianapolis, IN) and maintained at 37°C in 5% CO2.

### Selection and Characterization of Side Population Cells

To identify the SP among MM cells, the cells were resuspended at a concentration 1×10^6^ cells/mL in Hank’s balanced salt solution (HBSS) with 2% FBS and 10 mmol/L HEPES containing 5 µg/mL Hoechst 33342 dye and incubated for 60 min at 37°C with intermittent shaking. As a negative control, SP cells were preincubated with 100 µmol/L verapamil; 50 µmol/L reserpine, an ABC transporter inhibitor. At the end of the incubation, the cells were washed with ice-cold Phosphate Buffered Saline (Dulbecco’s PBS without calcium and magnesium, PBS(−)), after which they were centrifuged and resuspended in the HBSS buffer. To gate only viable cells, Propidium iodide solution (PI; Sigma Aldrich, St. Louis, MO; final concentration = 2 µg/mL) was added to the cells. The cells were then filtered through a 70-µm filter to obtain a single cell suspension, which was analyzed and sorted on a Moflo cell sorter (Dako, Glostrup, Denmark). The Hoechst 33342 dye was excited at 357 nm and its fluorescent emission was measured at 402–446 nm and 650–670 nm. After sorting, the SP cell fractions were analyzed using a UV laser flow cytometer (Dako) and found to be >98% pure. All experiments performed at least three times.

### Colony-forming Cell Assay

Cellular potential for clonogenicity of SP and MP cells of myeloma cell lines was determined using colony-forming cell (CFC) assay. Briefly, 2×10^3^ sorted SP and MP cells were plated in duplicate cultures containing one mL RPMI1640 with 1% methylcellulose, 30% FBS, 10-4 M 2-mercaptoethanol (Methocult; Stem Cell Technologies, Vancouver, BC, Canada), plus a cocktail of growth factors (50 ng/mL rh stem cell factor, 10 ng/mL rh GM-CSF, 10 ng/mL rh IL-3, and 3 U/mL erythropoietin). The cells were plated into 6-well tissue-culture plates, and after 10 days of incubation at 37°C in a ≥95%- humidified atmosphere containing 5% CO2. We used for scoring the number of colonies an inverted microscope with 4×, 10× and 20× planar objective followed standard criteria.

### XTT Assays

Cell viability assays were performed using an “*In Vitro* Toxicology Assay Kit, XTT based” according to manufacturers’ protocol (Sigma-Aldrich Co, St. Louis, MO).

### Fluorescence Immunophenotyping Assay

Cell lines were washed twice with PBS (−) and stained with PE/or FITC-conjugated mouse anti-human CD20, CD27, CD38 and CD138 antibodies (Beckman Coulter, Fullerton, CA) in PBS containing 2% BSA (Sigma, St Louis, MO) for 30 min at 4°C.

### *In vivo* Transplantation to NOG Mouse

5×10^2^–5×10^5^ SP or MP (from RPMI 8226) cells were injected subcutaneously into the right or left side of the body of 6–8 week–old female NOD/Shi-scid IL-2γnul (NOG) mice (Central Institute for Experimental Animals, Kawasaki, Japan) [27]. Similarly, 5×10^5^ SP or MP (from AMO1, KMS-12-BM and KMS-11) cells were injected subcutaneously into NOG mice. The protocols for animal experimentation described in this paper were previously approved by the Animal Committee, Akita University (approval number: b-1-2301); The “Regulation for Animal Experimentation” of the University were completely adhered to in all subsequent animal experiments.

### Gene Expression Analysis

RNA was extracted using an Arcturus PicoPure RNA isolation Kit (Applied Biosystems, Foster City, CA). We analyzed gene expression using a CodeLink™ Bioarray (Applied Microarrays, Inc., Tempe, AZ, USA). The array scanner was a GenePic 4000B (Molecular Devices, Sunnyvale, CA, USA), and the analysis software was Array-Pro Analyzer Version 4.5 (Media Cybernetics, Inc., Bethesda, MD, USA).

### Real Time Quantitative PCR Analysis

Quantitative stem-loop reverse transcription (RT) was performed using a First-Strand cDNA Synthesis Kit (GE Healthcare, Buckinghamshire, UK), after which quantitative PCR for cDNA was performed using TaqMan gene expression assays (Applied Biosystems). TaqMan probes of *CCNB1 (Hs01030097_m1), EZH2 (Hs01016789_m1), TOP2A (Hs00172214_m1), CDC2 (Hs00938777_m1), CDC20 (Hs00415851_g1), CDC25C (Hs00156411_m1), ASPM (Hs00411505_m1), AURKB (Hs00177782_m1), BIRC5 (Hs00220565_m1), UBE2D3 (Hs00704312_m1), PSMA5 (Hs00936004_m1), EPC1 (Hs00228677_m1)* and *GAPDH (Hs02758991_g1)* were purchased from Applied Biosystems. Messenger RNA (mRNA) was separately normalized with GAPDH, and the relative expression level of specific mRNA was presented by 2^–ΔΔCt^.

### Western Blot Analysis

Western blotting was carried out using standard methods. Antibodies against, p-Hist.H3, CDC2, p-CDC2 (T15), CyclinB1, p-Wee1 (ser642), Aurora B, p-Aurora B (Thr232) EZH2, and GAPDH were all purchased from Cell Signaling Technology (Danvers, MA, USA). Tubulin was purchased from Thermo scientific (Fremont, CA, USA). Cell monolayers were washed with PBS and lysed in RIPA buffer (50 mM Tris-HCL (pH 7.0), 1.0%NP-40, 0,1% dexycholic acid, 30 mM Na3VO4, 11 mM PMSF). Lystates were cleared by centrifugation and quantified using a Nanoorange Kit (Invitrogen). 15 µg total protein per lane was resolved by sodium dodecyl polyacrylamide gel electrophoresis (SDS-PAGE) in pre-cast gradient (4–15%) gels (Bio-Rad) at 120V for 60 minutes. Protein was transferred from gel to Immunobilon-P transfer membrane for 30 minutes, blocked overnight with milk/PBST and blotted with HRP-conjugated anti-mouse secondary antibody for 1 hour at room temperature. Tubulin of GAPDH was detected anti-human tubulin-HRP (1∶2000). Membranes were incubated with ECL plus (GE Healthcare) for 5 minutes and exposed to films for 15–60 seconds.

### Chemicals

Bortezomib (Vercade®) and VX-680 (MK-0457) were purchased from LC laboratories (New Boston Street Woburn, Canada). Dexamethasone (Sigma, Tokyo, Japan).

### Immunofluorescence Staining of p-Hist.H3

Cell lines were treated for 24 h with 0.1–10 µM VX-680 or 1–100 nM bortezomib, then fixed and permeabilized for 15 min with 4% formaldehyde in PBS(−), and blocked for 60 min in 5% normal goat serum in PBS(−). After washing, the cells were incubated for 1 h each at room temperature with anti-p-histone H3 antibody (Cell Signaling) diluted 1∶200 in PBS(−) Triton, followed by Alexa Fluor 488 goat anti-rabbit IgG (Invitrogen). After washing again, the cells were counterstained by incubation for 5 min with 1 mg/ml DAPI in PBS(−). Images were obtained using a fluorescence microscope (IX71; Olympus, Tokyo, Japan). The mitotic index was calculated by dividing the number of p-Hist.H3-positive cells by the total number of exposed cells (DAPI-positive cells). At least 100 cells were scored per low-power field, and the cells were counted over three fields. The experiment was performed in triplicate.

## Results

### SP Cells in MM Cell Lines Expressing CD138 Antigen

We screened for SP cells in human MM cell lines, based on their ability to export Hoechst 33342 dye. Flow cytometry revealed SP cells in the tail region of the dot plot. Fig. S1A depicts SP cells from the RPMI 8226, KMS-12-BM, AMO1 and KMS-11 lines: low-level accumulation of intracellular Hoechst 33342 is characteristic of SP cells (left panel), and the population disappears when the cells are treated with an ABC transporter inhibitor such as verapamil (100 µM; middle panel) or reserpine (50 µM; right panel). In fact, verapamil and reserpine reduced detection of SP cells in all cell lines examined with no significant cytotoxicity (Fig. S2). Fig. S1B shows the SP and MP fractions (%) in 14 MM cell lines, with and without verapamil or reserpine treatment. We found that the SP fractions in all examined cell lines with a range from 0.1% (KMS-20) to 27% (RPMI 8226).

**Figure 1:**
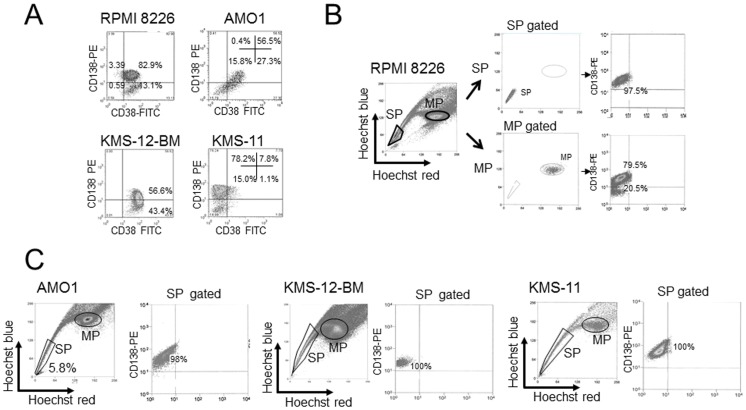
SP cells of myeloma cell lines are showing CD138. (A). Fluorescence immunophenotyping assay of indicated cells for CD38 and CD138. X-axis: FITC; Y-axis: PE. (B). Flow cytometric dot plots for SP and MP of RPMI 8226 cell line, representative and CD138-PE staining in SP and MP. (C). Flow cytometric dot plots for myeloma SP (AMO1, KMS-12-BM, KMS-11) and representative CD138-PE staining in SP are shown. All immunophenotyping assays were performed by three individual experiments and obtained similar results. %CD138+ cells of AMO1 SP were ranged from 96.6 to 98.0%, KMS-12-BM SP were from from 99.1 to 100%, KMS-11 SP were from 98.5 to 100%.

We first immunostained the cells for CD38, CD138, CD20 and CD27 to characterize the immunophenotypes of RPMI 8226, AMO1, KMS-12-BM and KMS-11 cell line (Fig. S3). We found that CD138 expression was heterogeneous, and there was a subpopulation of CD138^−^ cells (Fig. 1A). To compare CD138 expression in RPMI 8226 of SP and MP cells, we examined cells for Hoechst 33342 and CD138-PE staining, and found that nearly all SP cells (96.3–97.5%) were CD138^+^, while MP cells could be CD138^+^ (76.5–79.5%) or CD138^−^ cells (20.5–23.5%) (Fig. 1B). We next repeated these experiments using phenotypically distinct MM cells such as AMO1 (CD20^−^,CD38^+^, CD138^+^), KMS-12BM (CD20^+^, CD38^+^, CD138^+^) and KMS-11 (CD20^−^,CD38^−^, CD138^+^) cells. We found that these cell lines also showed heterogeneous pattern of CD138 expression (Fig. 1A). However, of note, almost all (>98%) SP cells from these cell lines also expressed CD138^+^ (Fig. 1C). Thus our results suggest that MM SP cells are expressing CD138.

### Clonogenic MM SP Cell Showing High Tumorigenic Potential and a Self-renewal Capability

To assess the clonogenicity of the SP, we performed CFC assays with SP and MP cells sorted from RPMI 8226, AMO1 and KMS-12-BM lines. After 10 days, sorted SP cells of the cell lines generated 12–14 (RPMI 8226), 5–12 (KMS-12-BM), and 10–16 (AMO1) times more colonies in comparison to MP cells (Fig. S4A). Importantly, the shape and size of the colonies differed significantly between the SP and MP cells. To examine whether SP cell possesses tumorigenic potential, we transplanted RPMI 8226 cells (5×10^5^ each) into immunodeficient (NOD/Shi-scid IL-2γnul) mice (NOG mice) and found that mice transplanted with SP cells showed much greater tumor development than those transplanted with MP cells (Fig. S4B left panel). Furthermore, SP cells could yield tumors with 5×10^2^–1×10^5^ transplantation into NOG mice (Fig. S4B right panel), although MP did not. Subcutaneous injection of SP cells of AMO1, KMS-12-BM and KMS-11 into NOG mouse readily reproduced the original tumor in the immunodeficient mice at day 31–44, whereas mice transplanted MP cells did not form tumors (AMO1) or smaller size of tumors (KMS-12-BM and KMS-11) (Fig. S4C). We termed these lesions “1^st^ generation” tumors.

**Figure 2:**
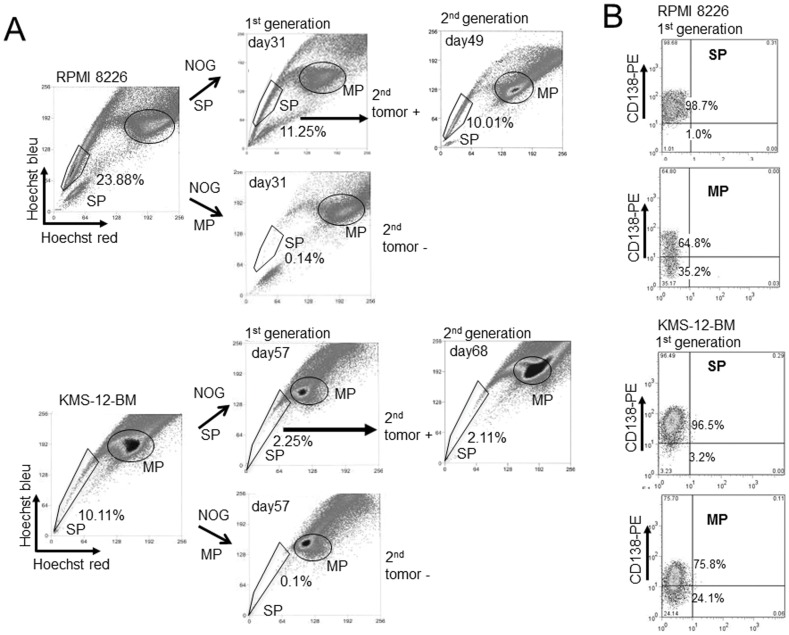
Serial transplantation of myeloma SP into NOG mice. (A). Serial transplantation of SP and MP cells into NOG mice. Sorted SP and MP cells collected 31 days (RPMI 8226, upper panel) or 57 days (KMS-12-BM, under panel) after subcutaneous inoculation into NOG mice were restained with Hoechst 33342 dye (shown as 1^st^ generation). Sorted SP cells from 1^st^ generation collected 49 days (RPMI 8226) or 68 days (KMS-12-BM) are also shown (shown as 2^nd^ generation). Shown is the % SP. (B). CD138 staining of SP and MP cells of 1^st^ generation from NOG mice. Data of RPMI 8226 (upper panel) and KMS-12-BM (under panel) are shown.

To test for self-renewal of SP and MP cells from the RPMI 8226 and KMS-12-BM cell lines, 1^st^ generation tumors were removed from NOG mice, after which the cells were subcultured and restained with Hoechst 33342. Then we conducted following serial transplantation experiments. We found that 1^st^ generation SPs could induce tumors (termed 2^nd^ generation tumors) but MPs did not induce tumor. 2^nd^ generation SPs also could yield tumors (termed 3^rd^ generation tumors). Furthermore, SP was showing the self-renewal capability; that is, they produced both an SP and MP, whereas MP produced only more MP (Fig. 2A). For instance, after 31 (RPMI 8226) or 57 (KMS-12-BM) days from the original transplantation, cultured SP cells produced significant fractions of both SP and MP cells, whereas MP cells produced only more MP cells (1^st^ generation). SP cells (1×10^5^) from the 1^st^ generation were further transplanted into NOG mice and again produced tumors (2^nd^ generation), which contained both SP and MP cells. SP cells (1×10^5^) from the 2^nd^ generation were then transplanted into NOG mice, and we confirmed the onset of 3^rd^ generation tumors. 1×10^5^ MP cells from the 1^st^ generation were also transplanted into NOG mice but did not yield 2^nd^ generation tumors.

In addition, CD138 expression in the 1^st^ generation of SP and MP cells in RPMI 8226 and KMS-12-BM were examined and found that nearly all SP cells expressed CD138, whereas the MP included both CD138^+^ and CD138^−^ cells (Fig. 2B).

### MM SPs Showing Cell Cycle Regulation

**Figure 3:**
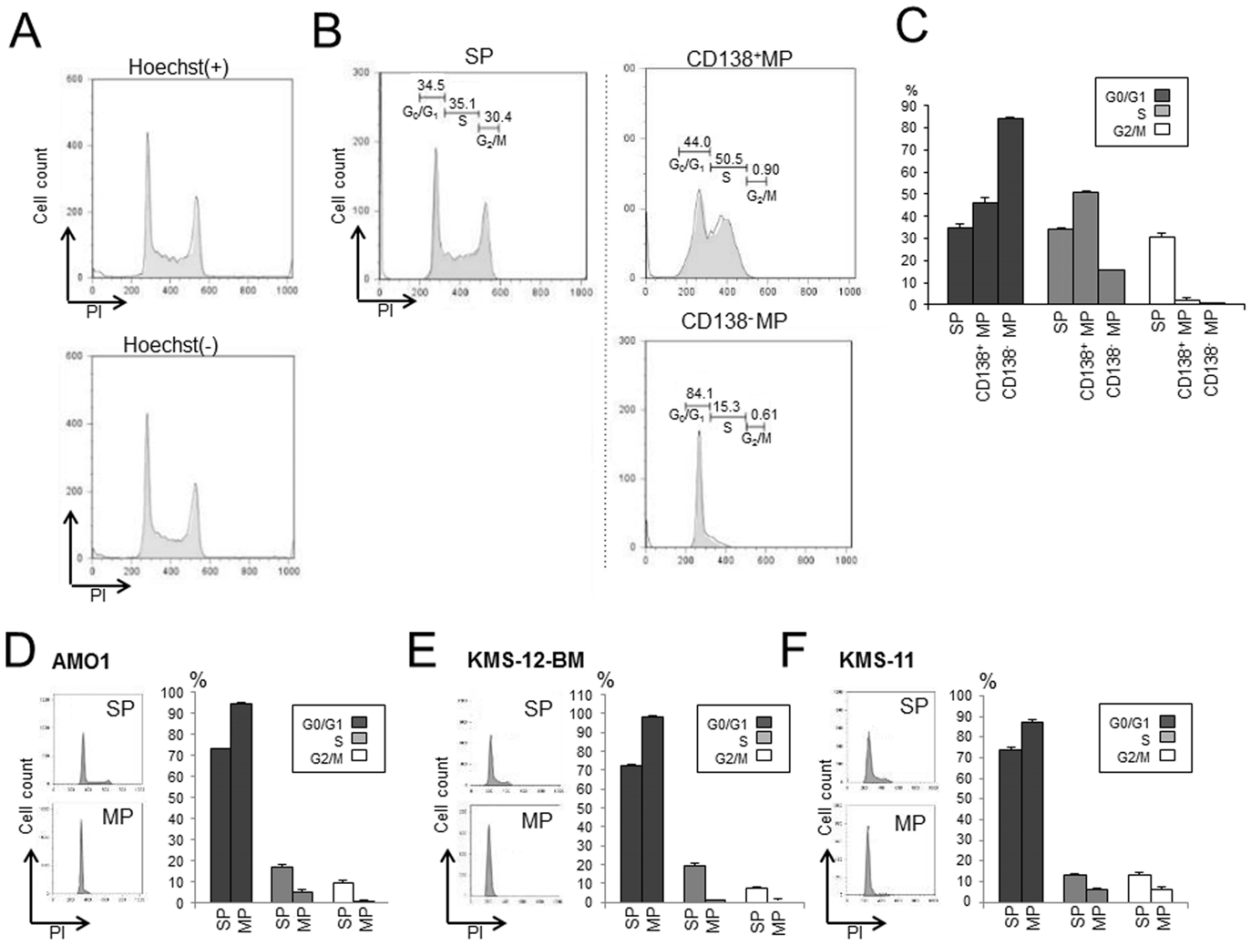
Cell cycle analysis of SP and MP of MM cell lines. (A). Cell cycle pattern of RPMI 8226 cell with and without Hoechst 33342 stain. (B). Cell cycle analysis of RPMI 8226 SP cells and CD138^+^ and CD138^−^ MP cells. % of each cell cycle phase is as follows; SP: G_0_/G_1_ 34.5%, S 35.1%, G_2_/M 30.4%, CD138^+^MP: G_0_/G_1_ 44.0%, S 50.5%, G_2_/M 0.90%, CD138^−^MP: G_0_/G_1_ 84.1%, S 15.3%, G_2_/M 0.61%. (C). G_0_/G_1_, S, and G_2_/M fractions (%) among SP, CD138^+^ MP and CD138^−^ MP cells in RPMI 8226. Bars are means ± SD of three independent experiments. (D–F). Cell cycle analysis of SP and MP cells from the AMO1 (D), KMS-12-BM (E) and KMS-11 (F) cell lines. Cell cycle patterns in SP and MP cells from the indicated lines are shown beside bar graphs of %SP and %MP. Bars are means ± SD of three independent experiments.

We examined cell proliferation ability as well as cell cycle analysis of SP and MP cells for RPMI 8226 cell line. We first confirmed that the cell cycle patterns of Hoechst-stained RPMI 8226 cells did not differ from untreated control cells (Fig. 3A). Next we performed cell cycle analysis as shown in Fig. 2B. We then found that the cell cycle patterns of SP and MP cells clearly differed: SP cells showed cell cycle progression (G_1_-S-G_2_/M), whereas both CD138^+^ and CD138^−^ MP cells showed cell cycle arrest (CD138^+^ MP: S/G_2_ arrest; CD138^−^ MP: G_1_/S arrest) (Fig. 3B). Moreover, the cell cycle patterns of other myeloma cell lines (AMO1, KMS-12-BM and KMS-11) were similar to those of RPMI 8226 cells: SP cells showed cell cycle progression and MP cells showed G_1_/S (CD138^−^ MP) or S/G_2_ (CD138^+^ MP) arrest (Fig. 3C–F). Collectively then, our findings indicate that MM cell lines are CD138^+^, have a capacity for have high tumorigenic potential with higher proliferation abilities than MP cells.

### Detection of Candidate Genes for Targeting MM SP Cells

Because MM SP cells have a capacity for have high tumorigenic potential, to therapeutically target MM cells, it will be important to identify genes specifically expressed in the MM SP. To detect genes differently expressed between SP and MP cells, we carried out a genome wide gene expression analysis using RPMI 8226 and AMO1 cells. Our criteria of cut off the gene expression analysis of SP/MP are up regulated genes: >2.0 or downregulated genes : <0.5, respectively. This analysis revealed 41 genes that were commonly upregulated in SP cells and two genes that were commonly downregulated; no commonly up- or downregulated microRNAs were matched to the criteria (Table S1AB).

**Figure 4:**
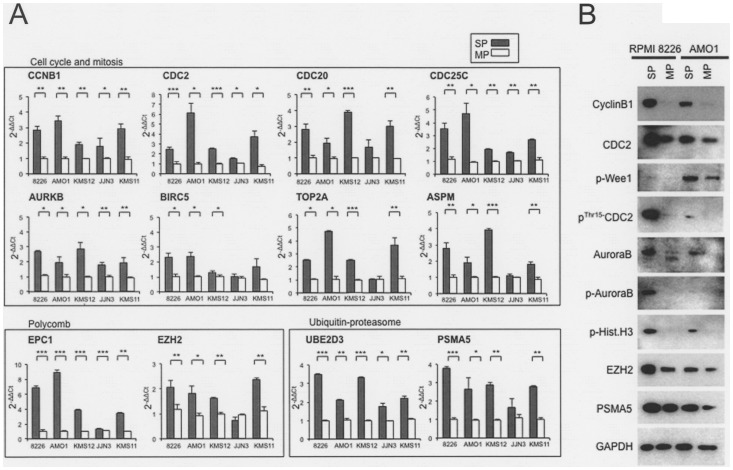
Real time quantitative PCR and western blot analyses of candidate markers of the SP in MM cells. (A). Real time quantitative PCR of cell cycle and mitosis related genes (*CCNB1,CDC2, CDC20, CDC25C, AURKB, BIRC5, TOP2A, ASPM),* Polycomb related genes (*EPC1, EZH2),* and ubiquitin-proteasome related gene (*UBE2D3* and *PSMA5)* against RPMI 8226, AMO1, KMS-12-BM, JJN3 and KMS-11 cells. Y-axis: gray and white bars depict 2^−ΔΔCt^ values for gene expression. Asterisks (*) indicate statistical significance: *0.01≤*P*<0.05, **0.001≤*P*<0.01, ***P<0.001. Bars are means ± SD of three independent experiments. (B). Western blot analysis of Cyclin B1, CDC2, p-WEE1, p-CDC2, Aurora B, p-Aurora B, p-Hist.H3, EZH2, PSMA5 and GAPDH in SP and MP against RPMI 8226 and AMO1 cell lines.

Up regulated are genes coding for proteins associated with cell cycle and mitosis (e.g. *CCNB1, CDC2, CDC25C, CDC20, CLK1, BIRC5, CENPE, SKA1, TPX2, NUSAP1, ASPM, TOP2A*, *AURKB, KIF15, KIF11, KIF2C, GTSE1*), polycomb (e.g. *EPC1*, *EZH2*), ubiquitin-proteasome related genes (e.g. *UBE3C, UBE2D3*) [28]–[30]. To confirm the gene expression levels, we conducted Real time quantitative PCR for twelve candidate genes of these genes using SP and MP cells from the RPMI 8226, AMO1, KMS-12-BM, JJN3 and KMS-11 lines (Fig. 4A). We found that in at least four of the five lines, ten genes were significantly more strongly expressed in SP than MP cells, and SP cells from three lines showed significantly stronger *BIRC5* expression. Moreover, Western blot analysis confirmed that cell cycle and mitosis-, proteasome- and polycomb-related proteins were all more strongly expressed in SP than MP cells from the RPMI 8226 and AMO1 lines (Fig. 4B). In addition, commonly downregulate genes (*AVEN* and *CDC42P3)* in RPMI 8226 and AMO1 (Table S1B) are also validated for the five cell lines. However, because there was no significant difference between SP and MP (data not shown), we did not further analysis for these genes.

We also separately analyzed aforementioned genes in SP, CD138^+^ MP and CD138^−^ MP cells from the RPMI 8226 and AMO1 lines. We found that the expression levels of the eight genes were significantly higher in SP cells than in either CD138^+^ or CD138^−^ MP cells (Fig. S5). Similarly, expression of these genes was higher in MM SP cells than in CD138^+^ or CD138^−^ MP cells. This suggests that comparison of gene expression between CD138^+^ SP and CD138^+^ MP cells might be a useful way to evaluate genes specifically expressed in primary MM SP cells.

### Real Time Quantitative PCR of Candidate Genes in SPs from Primary MM Samples

**Figure 5:**
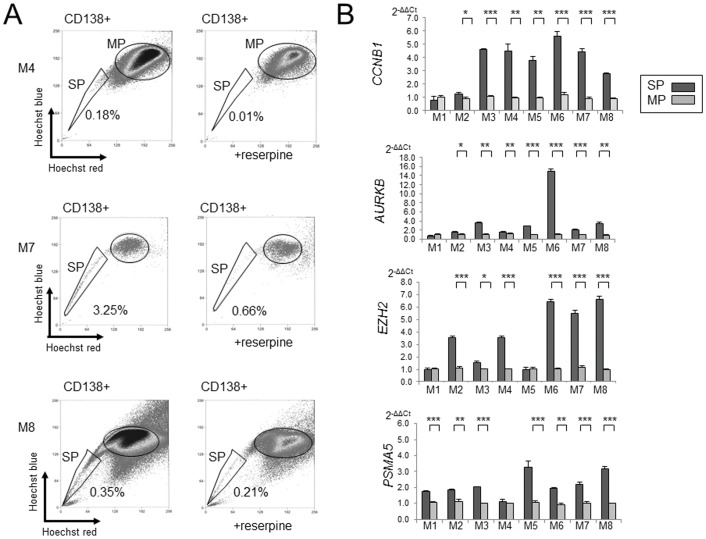
SP analysis in primary MM samples and gene expression analysis. (A). SP of primary samples (M4, M7 and M8). Left panel: cells obtained through bone marrow aspiration gated for CD138^+^ with and without 50 µM reserpine. SP fractions (%) are shown beside the SP gates surrounded by black lines. (B). Real time PCR analysis of eight samples of MM primary tumor cells. Shown bar graphs are *CCNB1, EZH2, AURKB* and *PSMA5* in SP and MP cells of indicated five myeloma cell lines. Y-axis: gray and white bars depict 2^−ΔΔCt^ values for gene expression. Asterisks (*) indicate statistical significance: *0.01≤*P*<0.05, **0.001≤*P*<0.01, ***P<0.001. Bars are means ± SD of triplicate samples.

Data obtained from our MM cell lines (Fig. 1) suggest that primary MM SP cells might also be identified based on CD138 positivity. We attempted to collect SP cells from eight primary samples (Table 1, Fig. 5A). The cells were then stained with both Hoechst 33342 and CD138-PE, and CD138^+^ SP and MP cells were collected and subjected to RNA extraction. We successfully sorted CD138^+^ SP cells from the eight samples, collecting from 1,500 to 50,000 SP and MP cells from each sample. The percentages of CD138^+^ SP cells ranged from 0.15% to 3.25%, while the percentages of CD138^−^ SP cells ranged from 0.37% to 2.93%. Addition of reserpine (50 µM) resulted in a 40% to 100% reduction in the size of the SP.

We performed Real time quantitative PCR analysis for four candidate genes of cell cycle and mitosis (*CCNB1* and *AURKB*), polycomb (*EZH2*) and ubiquitin-proteasome related (*PSMA5*) using the eight primary samples. Almost all cases showed upregulation of these genes examined: We found that seven cases (87.5%) showed significantly higher expressions of *CCNB1, AURKB* and *PSMA5* than MP cells and six cases (75%) of SP showed significantly higher expression of EZH2 than MP cells (Fig. 5B).

These results demonstrate that the SP of CD138^+^ primary MM cells also shows significantly greater expression of cell cycle and mitosis (*CCNB1* and *AURKB*)-, polycomb (*EZH2*)- and proteasome (*PSMA*5)-related genes than the MP. These results suggest that upregulated genes of CD138^+^ SP cells might be identical between cell lines and primary samples.

Because we were able to detect targets expressed specifically in MM SP cells (e.g., G_2_/M-related, polycomb and proteasome-related genes), we examined the effects of an aurora kinase inhibitor (VX-680) [31] and a proteasome inhibitor (bortezomb) [32], [33] on RPMI 8226 and AMO1 SP cells, which express different ABC transporters (RPMI 8226 SP: *ABCG2*; AMO1 SP: *ABCB1*).

### Effects of Aurora kinase Inhibitor (VX-680) for Targeting MM SP Cells

**Figure 6:**
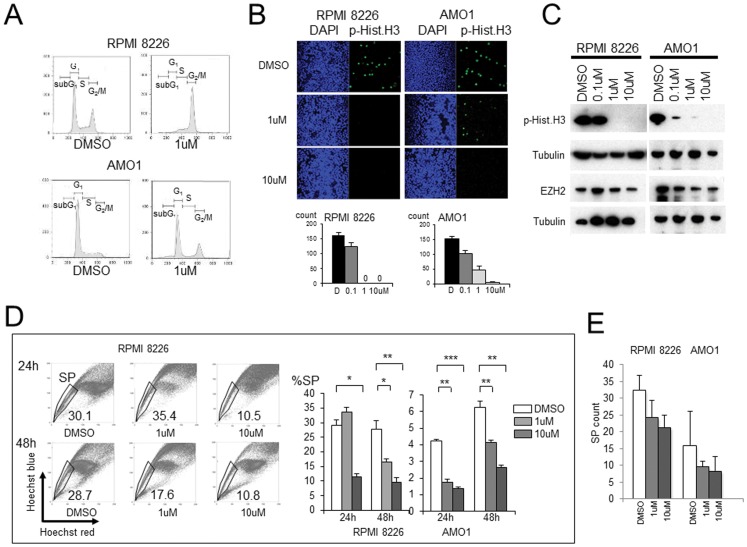
Effect of Aurora kinase inhibitor (VX-680) on myeloma SP cells. (A). Cell cycle analysis of RPMI 8226 and AMO1 cells exposed toVX-680 (1 µM). X-axis, PI; Y-axis, cell count. RPMI 8226+DMSO: subG1 1.1%, G_0_/G_1_ 47.3%, S 18.5%, G_2_/M 33.2%; RPMI 8226+VX-680 (1 uM): subG_1_ 1.7%, G_0_/G_1_ 3.4%, S 16.4%, G_2_/M 78.5%. AMO1+DMSO: subG_1_ 1.9%, G_0_/G_1_ 82.5%, S 22.4%, G_2_/M 12.9%; AMO1+VX-680 (1 uM): subG_1_ 8.4%, G_0_/G_1_ 54.8%, S 10.5%, G_2_/M 30.7%. (B). Detection of M phase cells among VX-680-treated MM cells. Upper panels: DAPI and p-Hist.H3 staining (green) of cells treated with DMSO, 1 µM, and 10 µM VX-680 (24 hr exposure). Under panels: bar graphs showing the numbers of M phase cells after treatment with the indicated concentration of VX-680 (24 hr exposure). (C). Western blot analysis of p-Hist.H3, EZH2 in RPMI 8226 (left panel) and AMO1 (right panel) cells; Tubulin is the control. (D). Flow cytometric analysis of RPMI 8226 SP cells. Dot plots of cells stained with Hoechst 33342 alone, Hoechst 33342 in the presence of 1 µM VX-680 or Hoechst 33342 in the presence of 10 µM VX-680. Left upper panels: 24 h exposure to VX-680; left lower panels: 48 h exposure to VX-680. Bar graphs of SP cell fractions (%) of indicated cells treated with VX-680 (DMSO, 1 µM, 10 µM) for 24 hr or 48 hr are also shown besides the flow cytometric analysis. DMSO is the control. Asterisks (*) indicate statistical significance: *0.01≤*P*<0.05, **0.001≤*P*<0.01, ***P<0.001. Bars are means ± SD of triplicate samples. (E). CFC assay. Colonies of SP by VX-680 (DMSO, 1 µM, 10 µM) for RPMI 8226 and AMO1 cell lines. Colony count was examined after 10 days from SP or MP distribution.

We initially conducted XTT assays with cells exposed to various concentrations of VX-680 to determine the IC^50^ (RPMI 8226∶6.5 µM and AMO1∶2 µM) (24 h exposure, data not shown). In subsequent experiments, cells were exposed to VX-680 for 24 or 48 h, as indicated. Among the VX-680-treated cells, large numbers were in G_2_/M phase and smaller numbers were in G_1_/S phase, which suggests G_2_/M arrest may have occurred in cells exposed to VX-680 (Fig. 6A).

Expression of Aurora B during the G_2_/M phase transition is tightly coordinated with histone H3 phosphorylation [34], [35]. We therefore immunostained for phospho-histone H3 (p-Hist.H3), an M-phase-specific marker, to determine whether there was a reduction in the number of M-phase cells. As expected, VX-680 induced cell cycle arrest at G_2_/M phase, with a corresponding reduction in the number of M-phase cells (Fig. 6B). The western blot in Fig. 6C shows the concentration-dependent reduction in p-Hist.H3 levels elicited by VX-680. Collectively, these results (Fig. 6A–C) suggest that VX-680 increases numbers of G_2_ cells while reducing the numbers of mitotic cells. We next examined expression of the polycomb proteins EZH2 in RPMI 8226 and AMO1 cells treated with different concentrations of VX-680. Cells exposed to VX-680 showed no reduction in the expression of these proteins, which suggests VX-680 may reduce SP cell proliferation by inhibiting their G_2_-M transition. Consistent with that idea, we found that VX-680 concentration-dependently reduced the %SP among RPMI 8226 and AMO1 cells (Fig. 6D). VX-680 thus appears to effectively reduce SP cell numbers by inhibiting cell proliferation. Further we examined effects of VX-680 for clonogenic abilities of SP against RPMI 8226 and AMO1 in presence (1 µM or 10 µM). Colonies of SP were slightly reduced in the presence of VX-680 but not yield significance (Fig. 6E).

### Effects of a Proteasome Inhibitor (bortezomib) Targeting MM SP Cells

**Figure 7:**
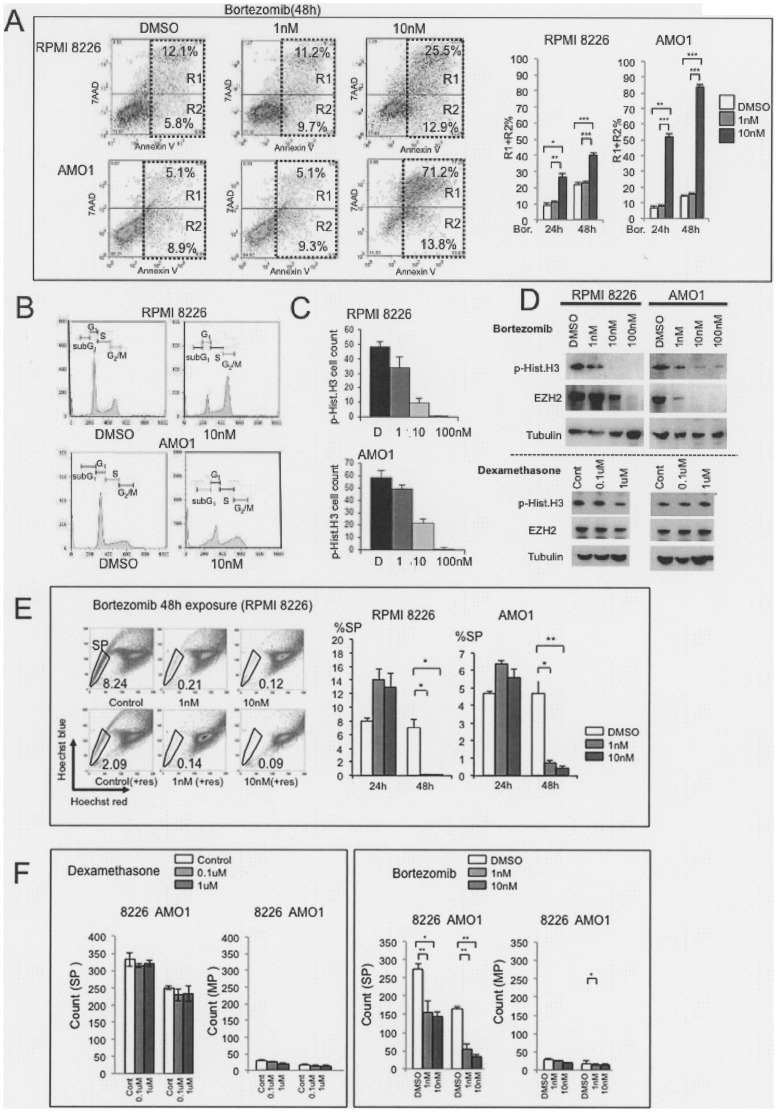
Reduction of the SP in MM cells treated with bortezomib. (A). Frequency of apoptosis of RPMI 8226 and AMO1. Left panels: dot plots showing the frequency of apoptosis at the indicated bortezomib (Bor.) concentrations (48 hr exposure). X-axis: cells stained with AnnexinV-PE. Y-axis: cells stained with 7-AAD. Right panels: bar graphs showing the % apoptotic cells (R1+R2) among examined cells treated with indicated concentration of bortezomib at 24 hr and 48 hr as indicated. Asterisks (*) indicate statistical significance: *0.01≤*P*<0.05, **0.001≤*P*<0.01, ***P<0.001. NS: not significant. (B). Cell cycle analysis RPMI 8226 and AMO1 treated with 10 nM bortezomib (24 hr). DMSO served as the control. RPMI 8226 control (+DMSO): subG_1_ 3.9%, G_0_/G_1_ 48.3%, S 19.2%, G_2_/M 28.6%; RPMI 8226+ bortezomib (10 nM): subG_1_ 5.0%, G_0_/G_1_ 20.1%, S 20.4%, G_2_/M 54.4%. AMO1 control (+DMSO): subG_1_ 1.3%, G_0_/G_1_ 58.4%, S 18.5%, G_2_/M 21.7%; AMO1+ bortezomib (10 nM): subG_1_ 14.8%, G_0_/G_1_ 31.6%, S 23.6%, G_2_/M 29.6% (C). Detection of M phase cells among bortezomib-treated (48 hr) myeloma cells. Bar graphs showing the numbers of M phase cells after treatment with DMSO, 1 nM, 10 nM and 100 nM bortezomib (24 hr exposure.) (D).Western blot analysis of p-Hist.H3 and EZH2 in RPMI 8226 (left panel) and AMO1 (right panel) cells after treatment with the indicated concentration of bortezomib and dexamethasone (48 hr). (E). Flow cytometric analysis of RPMI 8226 SP cells treated with bortezomib. Upper left panels: Dot plots of cells stained with Hoechst 33342 alone, Hoechst 33342 in the presence of 1 nM bortezomib for 48 h, or Hoechst 33342 in the presence of 10 nM bortezomib for 48 h. Lower left panels: cells treated as in the upper panels with 50 µM reserpine (shown as “res”). SP cell fractions (%) after treating RPMI 8226 and AMO1 cells are also shown besides the flow cytometric analysis. (F). CFC assay. Colonies of SP and MP by dexamethasone (left panel, Control (1 µl of 100% ethanol), 0.1 µM, 1 µM) and bortezomib (right pane, DMSO, 1 nM or 10 nM) for indicated cell lines. Asterisks (*) indicate statistical significance: *0.01≤*P*<0.05, **0.001≤*P*<0.01, ***P<0.001. Bars are means ± SD of three independent experiments.

The IC^50^ for bortezomib (48 h exposure) was 7 nM in RPMI 8226 cells and 4 nM in AMO1 cells (data not shown), and apoptosis assays showed that bortezomib concentration-dependently increased the incidence of apoptosis (Fig. 7A). We further examined its cell cycle effects. Bortezomib-treated cells included high numbers of G_2_/M phase cells and fewer G_1_/S phase cells (Fig. 7B), which suggests bortezomib induces G_2_/M arrest. This was confirmed by immunostaining for p-Hist.H3, which showed that bortezomib induced cell cycle arrest at G_2_/M phase, with a reduction in the numbers of M-phase cells (Fig. 7C). Moreover western blot analysis showed that bortezomib concentration-dependently reduced levels of p-Hist.H3, and EZH2, whereas dexamethasone did not (Fig. 7D). Thus bortezomib increases the number of G_2_ cells while decreasing the number of mitotic cells.

To determine the degree to which bortezomib reduced numbers of SP cells, we assessed the %SP after exposing cells to bortezomib for 24 or 48 h (Fig. 6E). We found that bortezomib markedly reduced the %SP among both RPMI 8226 and AMO1 cells after 48 h, though no reduction was seen after 24 h. After 48 h of treatment with 1 or 10 nM bortezomib, SP cell numbers were reduced by -99% among RPMI 8226 cells, and from 84% (1 nM) to 90% (10 nM) among AMO1 cells (Fig. 7E). These results suggest that bortezomib reduces SP cell numbers by inhibiting proliferation through inhibition of G_2_-M transition, *in vitro*. The effects of bortezomib on clonogenicity were examined using the RPMI 8226 and AMO1 SPs in the presence (1 nM or 10 nM) or absence (DMSO) of bortezomib (Fig. 7F right panel). SP cell colonies were significantly reduced in the presence of bortezomib. However there is no reduction of SP colonies in the presence of dexamethasome (Fig. 7F left panel). The results demonstrate that bortezomib can reduce the clonogenicity of SP among RPMI 8226 and AMO1 cells. This might be because bortezomib has a broader range of targets than VX-680; for example, bortezomib reduced expression not only of p-Hist.H3 but also EZH2.

## Discussion

SP cells are an enriched source of cancer-initiating cells with stem-like properties and have been identified in a variety of cancers, including MM [8]–[18]. The SP cells from the myeloma cell lines we examined showed clonogenicity and strong tumorigenicity. Moreover, when serially transplanted, myeloma SP cells yielded tumors in every generation, confirming that they possess a capacity for self-renewal. This population thus meets the criteria for cancer initiating cells [15], [16]. But although the myeloma SP likely contains true myeloma initiating cells, this population nonetheless appears heterogeneous. For example, we showed that myeloma SP cells express CD138 antigen, as previously reported [18]; however, CD138 is not a useful marker of myeloma-initiating cells because approximately 70–80% of MP cells also express the antigen. Further study will be required to identify specific antigens able to serve as markers of myeloma initiating cells within the SP from both cell lines and CD138^+^ primary samples.

Our aim in this study was to identify genes and gene products that were expressed specifically in the myeloma SP and so could serve as targets for anti-myeloma therapies. The genes we identified can be categorized into three groups: cell cycle and mitosis-related, polycomb-related, and proteasome-related. In the following, we will discuss the functions of representative genes and their products from these groups.

Aurora kinases (Aurora A, B and C) belong to a highly conserved family of mitotic serine/threonine kinases [36]–[39]. Among them, Aurora-B is a “chromosomal passenger” protein essential for the chromosome congression that regulates chromosome segregation through the control of microtubule-kinetochore attachment and cytokinesis [37]–[40]. Expression of Aurora B peaks during G_2_/M phase of the cell cycle, and its aberrant expression and activation occurs in a wide range of human tumors, leading to aneuploidy and tumorigenesis [6], [39], [40]. In addition, overexpression of kinase-inactive Aurora-B disrupts kinetochore-microtubule interactions, cleavage furrow formation and cytokinesis, leading to polyploidy in myeloma cells [6], [7], [40]. It is therefore conceivable that an aurora kinase inhibitor such as VX-680 would reduce numbers of myeloma SP cells. In our study, *AURKB* was strongly expressed in SP cells from both myeloma cell lines and primary myeloma samples. When we assessed the ability of VX-680 to target myeloma SP cells, we found that it effectively reduced SP numbers in myeloma cell lines. However VX-680 did not completely eliminate the population, suggesting that use of an aurora kinase inhibitor alone would be therapeutically insufficient. Instead, its use in combination with one or more agents targeting other molecules would be required for complete elimination of the myeloma SP.

*EZH2* and *EPC1* are two polycomb-related genes upregulated in the myeloma SP. EZH2 is a component of PRC2 (polycomb repressive complex 2) and represents one of two classes of polycomb-group (PcG) proteins. It is known that polycomb proteins play crucial roles in aggressive tumor growth and cancer stem cell maintenance [41], and that the EPC1-E2F6 complex interacts with EZH2 to contribute to cell proliferation [42]. It is thus plausible that interaction between *EPC1* and *EZH2* within myeloma SP cells contributes to their proliferation as well as to cancer stem cell maintenance. Consistent with that idea, *EZH2* is reportedly overexpressed in aggressive MM [6], [40]. In the present study, we for the first time demonstrated that the proteasome inhibitor bortezomib reduces both p-Hist.H3 and EZH2. By contrast, aurora kinase inhibition alone did not reduce EZH2 enough to diminish the clonogenicity of SP cells *in vitro*. This suggests bortezomib reduces myeloma SP cell clonogenicity by acting on both Aurora B and EZH2.

The naturally occurring and synthetic inhibitors of the ubiquitin-proteasome pathway include lactcystin, peptide aldehydes and boronic acid peptide [43]. The dipeptide boronic acid analogue bortezomib was the first proteasome inhibitor to be used as an anticancer agent in a clinical setting, and thanks to its potent, highly selective and reversible inhibition of proteasome activity [43], bortezomib administration is now the standard of care for newly diagnosed MM patients [5], [32], [33]. We found that the proteasome subunit gene *PSMA5* is upregulated in myeloma SP cells. The 26S proteasome consists of the cylindrical 20S proteasome, which is the proteolytic core particle, and the 19S regulator [44]. Structurally, the 20S proteasome consists of four stacked rings, each with seven distinct subunits. Its two outer rings are identical and composed entirely of α subunits (called PSMAs) [44], [45]. The 20S proteasome is the major proteolytic enzyme complex involved in intracellular protein degradation. Its broad substrate spectrum includes cell cycle regulators, signaling molecules, tumor suppressors and transcription factors [44], [45]. *PSMA5* encodes one of the α subunits of the 20S proteasome and Zhu and coworkers recently demonstrated that knocking down proteasome genes, including *PSMA5*, could negatively impact the inhibitory effects of bortezomib on growth [46], suggesting *PSMA5* transcription could be a key target for anti-myeloma SP therapy.

In summary, we found that MM SP cells exhibit strong tumorigenicity *in vivo*, and that they more strongly express cell cycle- and mitosis-related, polycomb-related, and ubiquitin-proteasome-related genes than do non-SP cells. Moreover, bortezomib has the ability to downregulate expression of these targets. Our approach could be useful for screening new agents with which to target a cell population possessing strong tumor initiating potential in MM.

## Supporting Information

Figure S1 **Detection of SP cells in a panel of MM cell lines.** (A). Representative flow cytometric dot plots for SP analysis. Dot plots show MM cells (RPMI 8226, KMS-12-BM, AMO1 and KMS-11) stained with Hoechst 33342 alone (left), Hoechst 33342 in the presence of 100 µM verapamil (middle), and Hoechst 33342 in the presence of 50 µM reserpine (right). The SP fractions (%) are shown beside each SP gate. X-axis, Hoechst red fluorescence intensity; Y-axis, Hoechst blue fluorescence intensity; the gate distinguishes the SP fraction among MM cells. (B). SP and MP fractions (%) in 14 MM cell lines. % SP, % SP+100 µM verapamil, % SP+50 µM reserpine, and % MP are shown for each cell line. Symbols and bars are means and SDs of triplicate samples.
(TIF)

Figure S2 **Cell viability of Hoechst 33342 stained MM cell lines treated with verapamil and reserpine.** XTT assay show MM cells (RPMI 8226, AMO1, KMS-11, and KMS-12-BM) stained with control cells, cells treated with Hoechst 33342 alone, treated with Hoechst 33342 in the presence of 0, 100 and 200 µM verapamil (upper panel). Lower panel shows control cells, Hoechst 33342 in the presence of 0, 50 and 100 µM reserpine. Cells were treated with Hoechst 33342 for 60 min with and without verapamil or reserpine.
(TIF)

Figure S3 **Detection of phenotypes of myeloma cell lines.** Fluorescence immunophenotyping assay of RPMI 8226, AMO1, KMS-12-BM and KMS-11 cells.
(TIF)

Figure S4 **Clonogenicity, tumorigenicity capabilities of MM SP cells.** (A). Clonogenicity of SP and MP cells. Y axis is no of colonies of both SP and MM of RPMI8226, KMS-12-BM and AMO1 cells. (B). In vivo engraftment of SP or MP cells of RPMI 8226 cells in NOG mice. Left panel: tumor growth from implanted cells (5×105, n = 3 each); right panel, in vivo engraft of SP (5×102, 1×103, 5×103, 1×104, 5×104, 1×105, n = 2 each) in NOG mouse. “+” indicate “scarified”. X-axis, days from implantation; Y-axis, tumor volume. (C). In vivo transplantation of MM cells into NOG mice. In vivo engraft of SP (5×105, n = 3) and MP (5×105, n = 3) of AMO1, KMS-12BM and KMS-11 in NOG mice. X axis: days from implantation; Y axis: tumor volume.
(TIF)

Figure S5 **Real time quantitative PCR analysis of candidate genes against SP, CD138+ MP and CD138- MP in RPMI 8226 and AMO1.** Real time quantitative PCR analysis of CCNB1, CDC2, CDC20, AURKB, ASPM, TOP2A, EZH2 and PSMA5 expression in SP, CD138+ MP and CD138- MP cells from the RPMI 8226 (dark gray) and AMO1 (right gray) lines. Asterisks (*) indicate statistical significance: *0.01≤P<0.05, **0.001≤P<0.01, ***P<0.001. Bars are means ± SD of triplicate samples.
(TIF)

Table S1 Genes of SP showing higher (Table S1A) or lower (Table S1B) expression than MP. Genes SP/MP>2.0, Genes SP/MP<0.5 are listed respectively.
(XLS)

## References

[1] McKenna RW (2008) Plasma cell neoplasms. In: Swerdlow SH, Campo E, Harris NL, Jaffe ES, Pileri S, editors. World Health Organization Classification of Tumours of Haematopoietic and Lymphoid Tissues. Lyon: IARC Press. 200–213.

[2] Kyle RA, Rajkumar SV (2008) Multiple myeloma. Blood 111: 2962–2972. 18332230 10.1182/blood-2007-10-078022 PMC2265446

[3] Bataille R, Harousseau JL (1997) Multiple myeloma. N Engl J Med 336: 1657–1664. 9171069 10.1056/NEJM199706053362307

[4] Greipp PR, San MJ, Durie BG, Crowley JJ, Barlogie B, et al (2005) International staging system for multiple myeloma. J Clin Oncol 23: 3412–3420. 15809451 10.1200/JCO.2005.04.242

[5] Mitsiades CS, Davies FE, Laubach JP, Joshua D, San Miguel J, et al (2011) Future directions of next-generation novel therapies, combination approaches, and the development of personalized medicine in myeloma. J Clin Oncol 29: 1916–1923. 21482978 10.1200/JCO.2010.34.0760

[6] Chng WJ, Braggio E, Mulligan G, Bryant B, Remstein E, et al (2008) The centrosome index is a powerful prognostic marker in myeloma and identifies a cohort of patients that might benefit from aurora kinase inhibition. Blood 111: 1603–1609. 18006703 10.1182/blood-2007-06-097774

[7] Hose D, Rème T, Meissner T, Moreaux J, Seckinger A, et al (2009) Inhibition of aurora kinases for tailored risk-adapted treatment of multiple myeloma. Blood 113: 4331–4340. 19171872 10.1182/blood-2008-09-178350 PMC2700334

[8] Zhou S, Schuetz JD, Bunting KD, Colapietro AM, Sampath J, et al (2001) The ABC transporter Bcrp1/ABCG2 is expressed in a wide variety of stem cells and is a molecular determinant of the side-population phenotype. Nat Med 7: 1028–1034. 11533706 10.1038/nm0901-1028

[9] Challen GA, Little MH (2006) A side order of stem cells: the SP phenotype. Stem Cells 24: 3–12. 16449630 10.1634/stemcells.2005-0116

[10] Haraguchi N, Utsunomiya T, Inoue H, Tanaka F, Mimori K, et al (2006) Characterization of a side population of cancer cells from human gastrointestinal system. Stem Cells 24: 506–513. 16239320 10.1634/stemcells.2005-0282

[11] Szotek PP, Pieretti-Vanmarcke R, Masiakos PT, Dinulescu DM, Connolly D, et al (2006) Ovarian cancer side population defines cells with stem cell-like characteristics and mullerian inhibiting substance responsiveness. Proc Natl Acad Sci U S A 103: 11154–11159. 16849428 10.1073/pnas.0603672103 PMC1544057

[12] Ho MM, Ng AV, Lam S, Hung JY (2007) Side population in human lung cancer cell lines and tumors is enriched with stem-like cancer cells. Cancer Res 67: 4827–4833. 17510412 10.1158/0008-5472.CAN-06-3557

[13] Tavaluc RT, Hart LS, Dicker DT, El-Deiry WS (2007) Effects of low confluency, serum starvation and hypoxia on the side population of cancer cell lines. Cell Cycle 6: 2554–2562. 17912032 10.4161/cc.6.20.4911

[14] Chiba T, Miyagi S, Saraya A, Aoki R, Seki A, et al (2008) The polycomb gene product BMI1 contributes to the maintenance of tumor-initiating side population cells in hepatocellular carcinoma. Cancer Res 68: 7742–7749. 18829528 10.1158/0008-5472.CAN-07-5882

[15] Reya T, Morrison SJ, Clarke MF, Weissman IL (2001) Stem cells, cancer, and cancer stem cells. Nature 414: 105–111. 11689955 10.1038/35102167

[16] Jordan CT, Guzman ML, Noble M (2006) Cancer stem cells. N Engl J Med 355: 1253–1261. 16990388 10.1056/NEJMra061808

[17] Patrawala L, Calhoun T, Schneider-Broussard R, Zhou J, Claypool K, et al (2005) Side population is enriched in tumorigenic, stem-like cancer cells, whereas ABCG2+ and ABCG2− cancer cells are similarly tumorigenic. Cancer Res 65: 6207–6219. 16024622 10.1158/0008-5472.CAN-05-0592

[18] Jakubikova J, Adamia S, Kost-Alimova M, Klippel S, Cervi D, et al (2011) Lenalidomide targets clonogenic side population in multiple myeloma: pathophysiologic and clinical implications. Blood 117: 4409–4419. 21321360 10.1182/blood-2010-02-267344 PMC3099565

[19] Moore GE, Marbell JW, Woods LK, Morgan KT, Semple TV (1982) RPMI 8226, a human myeloma cell line: an update. Proc. Am. Assoc. Cancer Res 33: 126–129.

[20] Namba M, Ohtsuki T, Mori M, Togawa A, Wada H, et al (1989) Establishment of five human myeloma cell lines. In Vitro Cell Dev Biol 25: 723–729. 2768132 10.1007/BF02623725

[21] Shimizu S, Takiguchi T, Fukutoku M, Yoshioka R, Hirose Y, et al (1993) Establishment of a CD4-positive plasmacytoma cell line (AMO1). 7: 274–280. 8426482

[22] Gu ZJ, Costes V, Lu ZY, Zhang XG, Pitard V, et al (1996) Interleukin-10 is a growth factor for human myeloma cells by induction of an oncostatin M autocrine loop. Blood. 88: 3972–3986. 8916964

[23] Tagawa S, Doi S, Taniwaki M, Abe T, Kanayama Y, et al (1990) Amylase-producing plasmacytoma cell lines, AD3 and FR4, with der(14)t(8;14) and dic(8)t(1;8) established from ascites. Leukemia. 4: 600–605. 1697013

[24] Takahira H, Kozuru M, Hirata J, Obama K, Uike N, et al (1994) Establishment of a human myeloma cell line with growth-promoting activity for bone marrow-derived fibroblastoid colony-forming cells.Exp Hematol. 22: 261–266. 8112425

[25] Oh SJ, Ryu CK, Choi I, Baek SY, Lee H (2012) Chemotherapeutic candidate inducing immunological death of human tumor cell lines. Immune Netw. 12: 66–69. 10.4110/in.2012.12.2.66 PMC3382666 22740792

[26] Otsuki T, Yata K, Takata-Tomokuni A, Hyodoh F, Miura, et al (2004) Expression of protein gene product 9.5 (PGP9.5)/ubiquitin-C-terminal hydrolase 1 (UCHL-1) in human myeloma cells. Br J Haematol. 127: 292–298. 10.1111/j.1365-2141.2004.05205.x 15491288

[27] Ito M, Hiramatsu H, Kobayashi K, Suzue K, Kamata M, et al (2002) NOD/SCID/gamma(c)(null) mouse: an excellent recipient mouse model for engraftment of human cells. Blood 100: 3175–3182. 12384415 10.1182/blood-2001-12-0207

[28] Pines J, Hunter T (1991) Human cyclins A and B1 are differentially located in the cell and undergo cell cycle-dependent nuclear transport. J. Cell Biol 115: 1–17. 1717476 10.1083/jcb.115.1.1 PMC2289910

[29] Strausfeld U, Labbe JC, Fesquet D, Cavadore JC, Picard A, et al (1991) Dephosphorylation and activation of a p34cdc2/cyclin B complex in vitro by human CDC25 protein. Nature 351: 242–245. 1828290 10.1038/351242a0

[30] Stankunas K, Berger J, Ruse C, Sinclair DA, Randazzo F, et al (1998) The enhancer of polycomb gene of Drosophila encodes a chromatin protein conserved in yeast and mammals. Development 125: 4055–4066. 9735366 10.1242/dev.125.20.4055

[31] Harrington EA, Bebbington D, Moore J, Rasmussen RK, Ajose-Adeogun AO, et al (2004) VX-680, a potent and selective small-molecule inhibitor of the Aurora kinases, suppresses tumor growth in vivo. Nat. Med 10: 262–267. 10.1038/nm1003 14981513

[32] Mitsiades N, Mitsiades CS, Poulaki V, Chauhan D, Fanourakis G, et al (2002) Molecular sequelae of proteasome inhibition in human multiple myeloma cells. Proc Natl Acad Sci U S A 99: 14374–14379. 12391322 10.1073/pnas.202445099 PMC137891

[33] Hideshima T, Bradner JE, Wong J, Chauhan D, Richardson P, et al (2005) Small molecule inhibition of proteasome and aggresome function induces synergistic antitumor activity in multiple myeloma. Proc Natl Acad Sci U S A 102: 8567–8572. 15937109 10.1073/pnas.0503221102 PMC1150844

[34] Pascreau G, Arlot-Bonnemains Y, Prigent C (2003) Phosphorylation of histone and histone-like proteins by aurora kinases during mitosis. Prog Cell Cycle Res 5: 369–374. 14593731

[35] Crosio C, Fimia GM, Loury R (2002) Mitotic phosphorylation of histone H3: spatio-temporal regulation by mammalian Aurora kinases. Mol Cell Biol. 22(3): 874–85. 10.1128/MCB.22.3.874-885.2002 PMC133550 11784863

[36] Nigg EA (2001) Mitotic kinases as regulators of cell division and its checkpoints. Nat. Rev. Mol. Cell Biol 2: 21–32. 10.1038/35048096 11413462

[37] Carmena M, Earnshaw WC (2003) The celluler geography of Aurora kinases. Nat Rev. Mol.biol 4: 842–854. 10.1038/nrm1245 14625535

[38] Jordan P, Copsey A, Newnham L, Kolar E, Lichten M, et al (2009) Hoffmann E. Ipl1/Aurora B kinase coordinates synaptonemal complex disassembly with cell cycle progression and crossover formation in budding yeast meiosis. Genes Dev. 23: 2237–2251. 10.1101/gad.536109 PMC2751982 19759266

[39] Katayama H, Brinkley WR, Sen S (2003) The Aurora kinases: role in cell transformation and tumorigenesis. Cancer Metastasis Rev. 22: 451–464. 10.1023/a:1023789416385 12884918

[40] Chng WJ, Ahmann GJ, Henderson K, Santana-Davila R, Greipp PR, et al (2006) Clinical implication of centrosome amplification in plasma cell neoplasm. Blood 107: 3669–3675. 16373658 10.1182/blood-2005-09-3810 PMC1895774

[41] Suvà ML, Riggi N, Janiszewska M, Radovanovic I, Provero P, et al (2009) EZH2 is essential for glioblastoma cancer stem cell maintenance. Cancer Res 69: 9211–9218. 19934320 10.1158/0008-5472.CAN-09-1622

[42] Attwooll C, Oddi S, Cartwright P, Prosperini E, Agger K, et al (2005) A novel repressive E2F6 complex containing the polycomb group protein, EPC, that interacts with EZH2 in a proliferation-specific manner. J Biol Chem 280: 1199–1208. 15536069 10.1074/jbc.M412509200

[43] Adams J (2002) The proteasome: a suitable antineoplastic target. Nat Rev Cancer 4: 349–360. 10.1038/nrc1361 15122206

[44] Lupas A, Koster AJ, Baumeister W (1993) Structural features of 26S and 20S proteasomes. Enzyme Protein 47: 252–273. 7697124 10.1159/000468684

[45] Pickart CM, Cohen RE (2004) Proteasomes and their kin: proteases in the machine age. Nat Rev Mol Cell Biol 5: 177–187. 14990998 10.1038/nrm1336

[46] Zhu YX, Tiedemann R, Shi CX, Yin H, Schmidt JE, et al (2011) RNAi screen of the druggable genome identifies modulators of proteasome inhibitor sensitivity in myeloma including CDK5. Blood 117: 3847–3857. 21289309 10.1182/blood-2010-08-304022 PMC3083298

