# Supplementary figures and images for: Bortezomib Reduces the Tumorigenicity of Multiple Myeloma via Downregulation of Upregulated Targets in Clonogenic Side Population Cells
Source: PLoS One. 2013 Mar 4;8(3):e56954. doi: 10.1371/journal.pone.0056954 (PMC3587640; doi:10.1371/journal.pone.0056954)

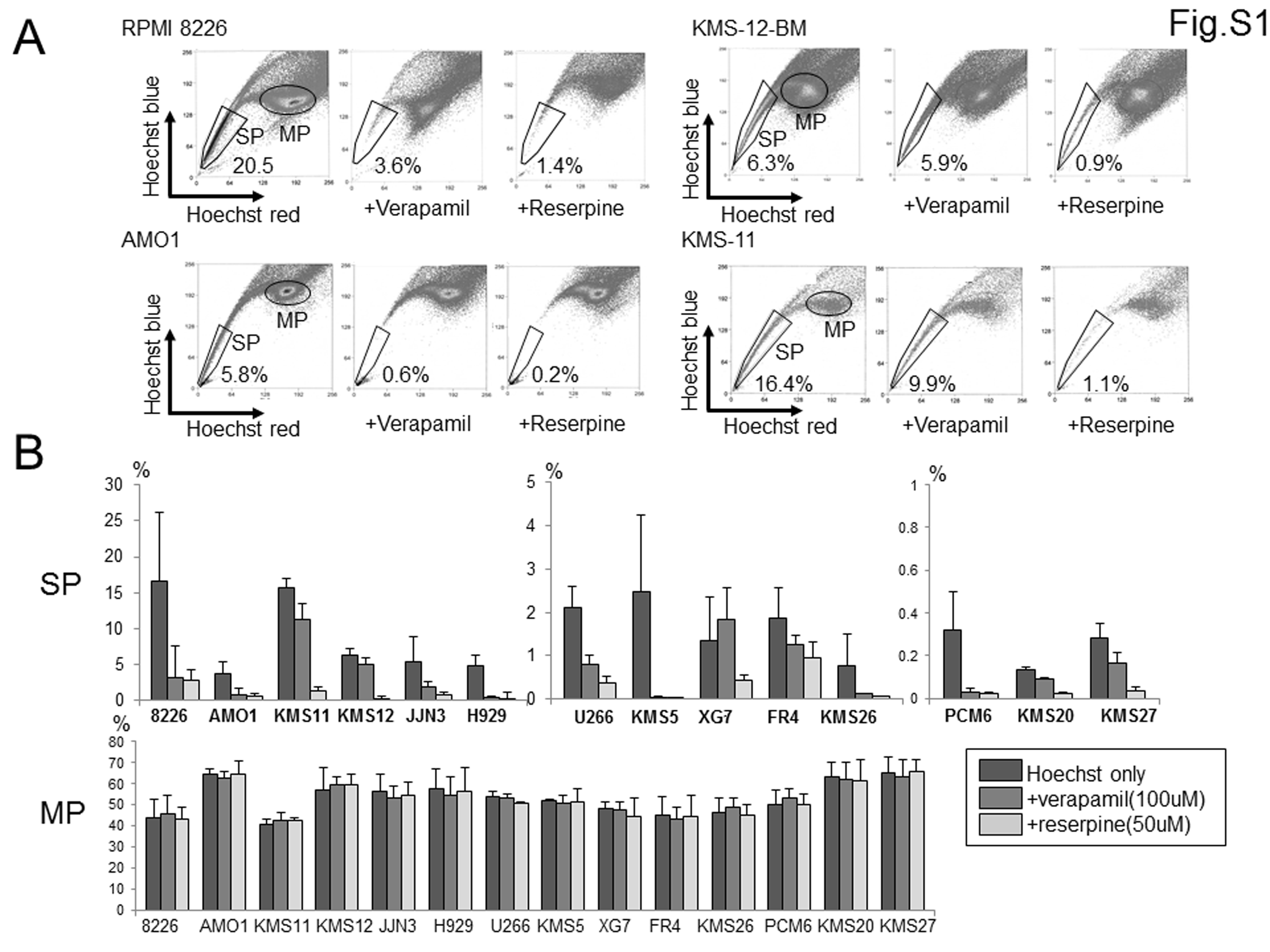

Supplement: Figure S1 — Detection of SP cells in a panel of MM cell lines. (A). Representative flow cytometric dot plots for SP analysis. Dot plots show MM cells (RPMI 8226, KMS-12-BM, AMO1 and KMS-11) stained with Hoechst 33342 alone (left), Hoechst 33342 in the presence of 100 µM verapamil (middle), and Hoechst 33342 in the presence of 50 µM reserpine (right). The SP fractions (%) are shown beside each SP gate. X-axis, Hoechst red fluorescence intensity; Y-axis, Hoechst blue fluorescence intensity; the gate distinguishes the SP fraction among MM cells. (B). SP and MP fractions (%) in 14 MM cell lines. % SP, % SP+100 µM verapamil, % SP+50 µM reserpine, and % MP are shown for each cell line. Symbols and bars are means and SDs of triplicate samples. (TIF) [file pone.0056954.s001.tif]

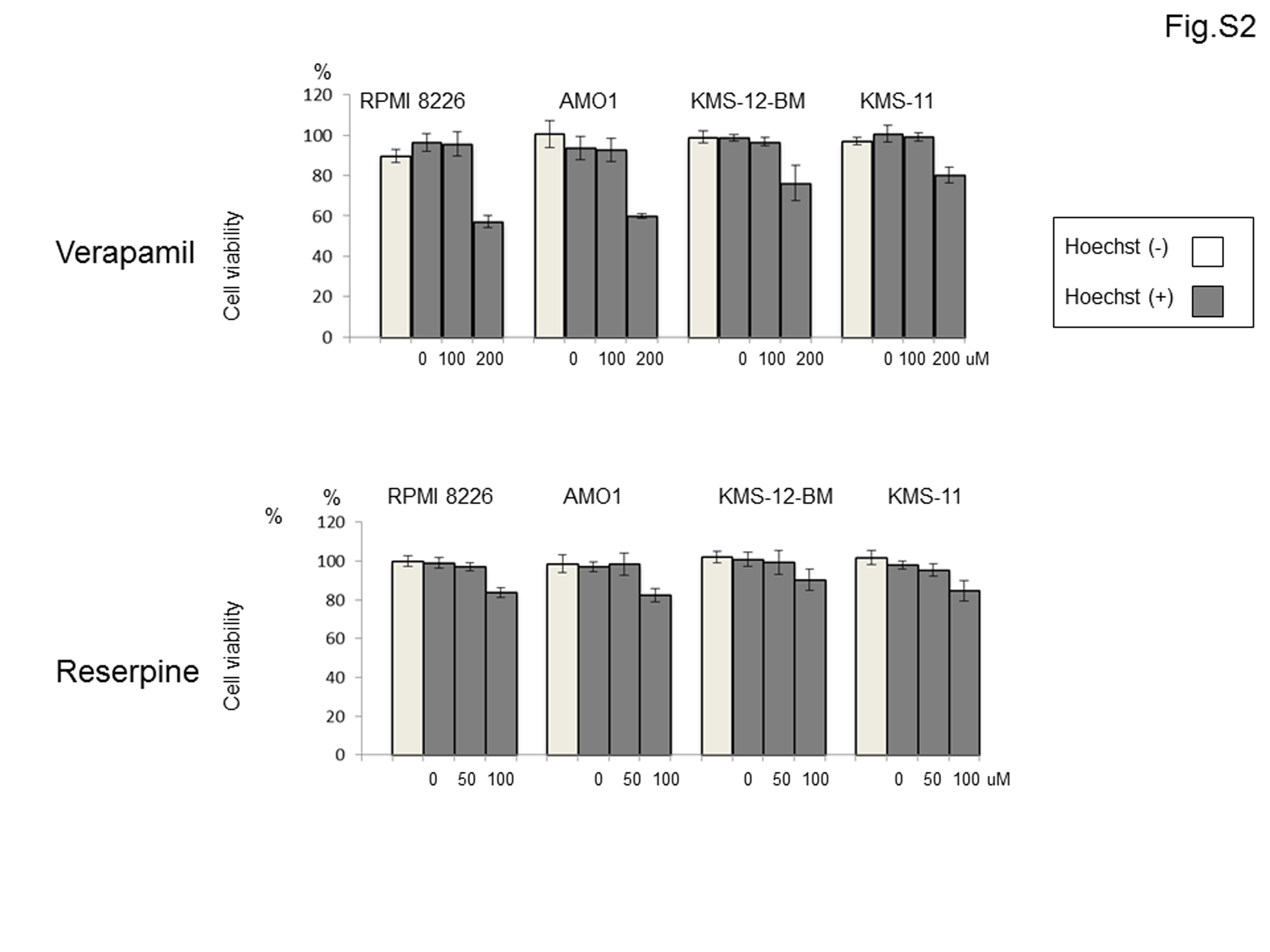

Supplement: Figure S2 — Cell viability of Hoechst 33342 stained MM cell lines treated with verapamil and reserpine. XTT assay show MM cells (RPMI 8226, AMO1, KMS-11, and KMS-12-BM) stained with control cells, cells treated with Hoechst 33342 alone, treated with Hoechst 33342 in the presence of 0, 100 and 200 µM verapamil (upper panel). Lower panel shows control cells, Hoechst 33342 in the presence of 0, 50 and 100 µM reserpine. Cells were treated with Hoechst 33342 for 60 min with and without verapamil or reserpine. (TIF) [file pone.0056954.s002.tif]

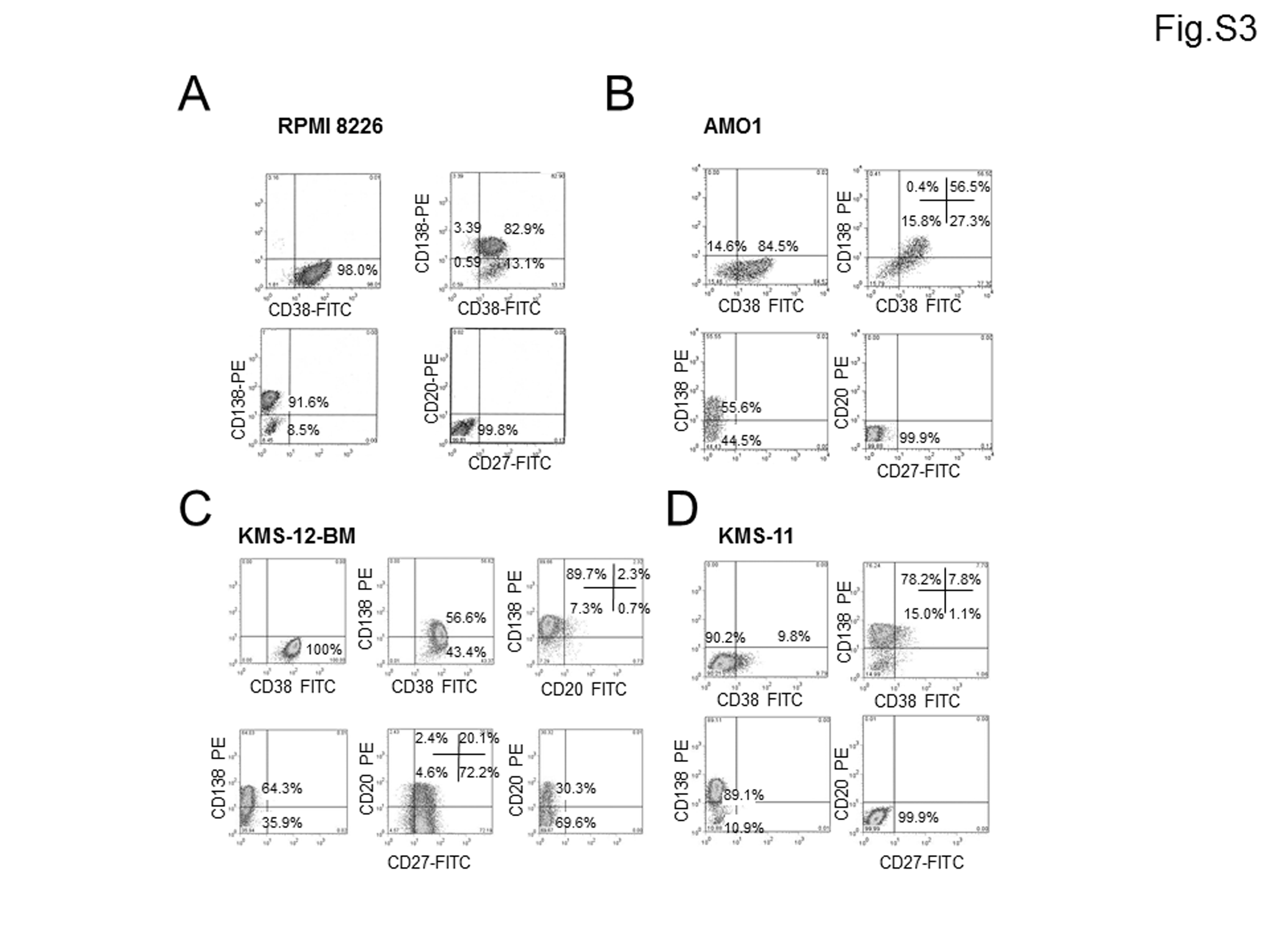

Supplement: Figure S3 — Detection of phenotypes of myeloma cell lines. Fluorescence immunophenotyping assay of RPMI 8226, AMO1, KMS-12-BM and KMS-11 cells. (TIF) [file pone.0056954.s003.tif]

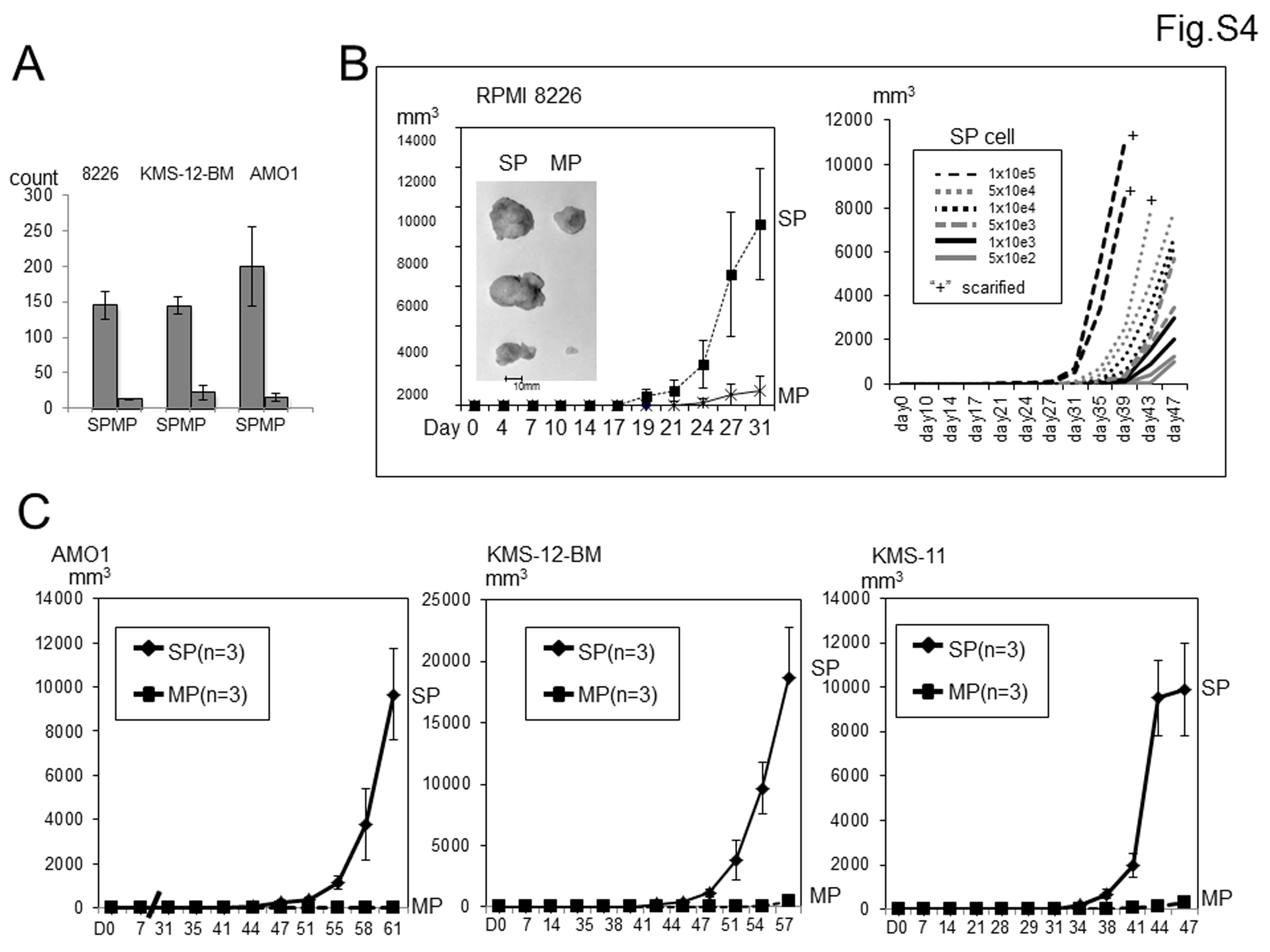

Supplement: Figure S4 — Clonogenicity, tumorigenicity capabilities of MM SP cells. (A). Clonogenicity of SP and MP cells. Y axis is no of colonies of both SP and MM of RPMI8226, KMS-12-BM and AMO1 cells. (B). In vivo engraftment of SP or MP cells of RPMI 8226 cells in NOG mice. Left panel: tumor growth from implanted cells (5×10^5^, n = 3 each); right panel, in vivo engraft of SP (5×10^2^, 1×10^3^, 5×10^3^, 1×10^4^, 5×10^4^, 1×10^5^, n = 2 each) in NOG mouse. “+” indicate “scarified”. X-axis, days from implantation; Y-axis, tumor volume. (C). In vivo transplantation of MM cells into NOG mice. In vivo engraft of SP (5×10^5^, n = 3) and MP (5×10^5^, n = 3) of AMO1, KMS-12BM and KMS-11 in NOG mice. X axis: days from implantation; Y axis: tumor volume. (TIF) [file pone.0056954.s004.tif]

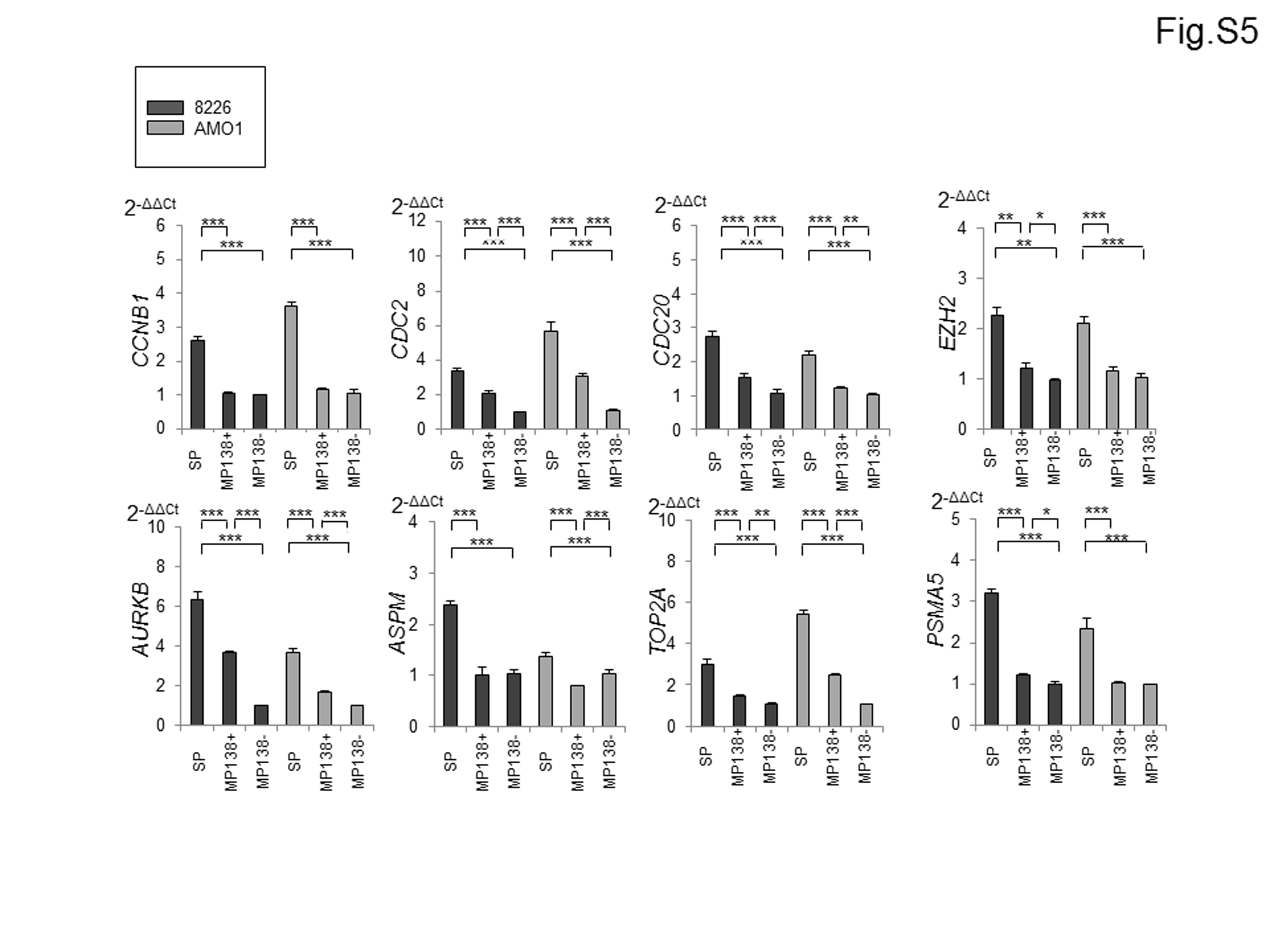

Supplement: Figure S5 — Real time quantitative PCR analysis of candidate genes against SP, CD138+ MP and CD138- MP in RPMI 8226 and AMO1. Real time quantitative PCR analysis of CCNB1, CDC2, CDC20, AURKB, ASPM, TOP2A, EZH2 and PSMA5 expression in SP, CD138+ MP and CD138- MP cells from the RPMI 8226 (dark gray) and AMO1 (right gray) lines. Asterisks (*) indicate statistical significance: *0.01≤P<0.05, **0.001≤P<0.01, ***P<0.001. Bars are means ± SD of triplicate samples. (TIF) [file pone.0056954.s005.tif]
